# Effect of sigmoidectomy in treating sigmoid colon cancer: A protocol of systematic review

**DOI:** 10.1097/MD.0000000000023914

**Published:** 2021-01-22

**Authors:** Hua-ping Hou, Pu-guo Gui

**Affiliations:** a Department of General Surgery, The First Hospital of Yulin, Yulin; b Department of General Surgery, Yangling Demonstration District Hospital, Xianyang, Shaanxi, China.

**Keywords:** effect, sigmoid colon cancer, sigmoidectomy

## Abstract

**Background:**

This study will assess the effect of sigmoidectomy in treating sigmoid colon cancer (SCC).

**Methods:**

This study will search the following databases from inception to the present: MEDLINE, EMBASE, Cochrane Library, CINAHL, PsycINFO, Scopus, OpenGrey, Chinese Biomedical Literature Database, and China National Knowledge Infrastructure. All electronic databases will be searched with no restrictions of language. Two researchers will independently handle all study selection, data extraction, and risk of bias, respectively. Any disparities between 2 researchers will be figured out by a third researcher through discussion. RevMan 5.3 software will be used for statistical analysis in this study.

**Results:**

This study will provide a high-quality synthesis of targeted outcomes to evaluate the efficacy and complications of sigmoidectomy in treating SCC.

**Conclusion:**

The results of this study will provide evidence to judge whether sigmoidectomy can benefit patients with SCC.

**Study registration on OSF:**

osf.io/dpxkg.

## 1 Introduction

Colon cancer (CC) is one of most common malignancy tumors and also the leading cause of cancer-related death globally.^[1]^ At initial stage, about 10% patients with CC have a primary tumor proceeding to adjacent tissues.^[2]^ Sigmoid colon is the final section of colon before rectum, and shaped like an “S.”^[3,4]^ Sigmoid colon cancer (SCC), a type of CC, occurs in the sigmoid colon section.^[3,5–7]^ Its symptoms are mostly mild or inconspicuous at the early stage, and thus are not easily to be detected.^[8]^ At the middle and late stages, it manifests as persistent abdominal discomfort, dull pain, bloating, constipation, and intestinal obstruction.^[9,10]^ Thus, it is very important to diagnose and treat at early stage.

Surgery is the principal treatment of SCC.^[11–13]^ A variety of previous clinical studies have reported to use sigmoidectomy for the treatment of SCC.^[14–24]^ However, there is still insufficient evidence-based medicine evidence to support sigmoidectomy for SCC. Therefore, this study will comprehensively and systematically assess the effect of sigmoidectomy in treating SCC.

## 2 Methods

### 2.1 Dissemination and ethics

We will plan to submit this study at a peer-reviewed journal or a relevant conference. No ethic approval document is needed because this study will not employ any individual data.

### 2.2 Study registration

We have registered on OSF (osf.io/dpxkg). It will be conducted according to the guidelines of Cochrane Handbook for Systematic Reviews of Interventions and the Preferred Reporting Items for Systematic Reviews and Meta-Analysis Protocol statement.^[25]^

### 2.3 Inclusion criteria for study selection

#### 2.3.1 Types of studies

All relevant randomized controlled trials (RCTs) of sigmoidectomy in treating SCC will be included without restrictions of language and publication status.

#### 2.3.2 Types of participants

Inclusion criteria for study participants will be all eligible patients who were diagnosed as SCC. No limitations will be applied in terms of country, ethnicity, and educational background.

#### 2.3.3 Types of interventions

In the experimental group, intervention to be utilized is sigmoidectomy alone.

In the control group, any treatment management could be used, such as radiotherapy, chemotherapy, but not sigmoidectomy.

#### 2.3.4 Type of outcome measurements

Outcomes include overall survival, pathological complete response, progression-free survival, recurrence-free survival, and any complication.

### 2.4 Search methods for the identification of studies

#### 2.4.1 Electronic databases search

We will search the following databases from inception to the present: MEDLINE, EMBASE, Cochrane Library, CINAHL, PsycINFO, Scopus, OpenGrey, Chinese Biomedical Literature Database, and China National Knowledge Infrastructure. No language and publication status limitations will be imposed to all above electronic databases. We will build a detailed search strategy for Cochrane Library in Table 1. In addition, we will also adapt similar search strategies to any other electronic databases.

**Table 1:** Search strategy of Cochrane Library.

Number	Search terms
1	MeSH descriptor: (sigmoid neoplasms) explode all trees
2	MeSH descriptor: (colonic neoplasms) explode all trees
3	((neoplasm^∗^) or (cancer^∗^) or (tumor^∗^) or (colon^∗^) or (sigmoid^∗^) or (colonic^∗^) or (intestinal^∗^) or (colorectal^∗^)):ti, ab, kw
4	Or 1-3
5	MeSH descriptor: (general surgery) explode all trees
6	((surgery^∗^) or (operation^∗^) or (sigmoidectomy^∗^) or (resection^∗^) or (laparoscopic^∗^) or (surgical procedure^∗^)):ti, ab, kw
7	Or 5–6
8	MeSH descriptor: (randomized controlled trials) explode all trees
9	((random^∗^) or (randomly^∗^) or (allocation^∗^) or (placebo^∗^) or (blind^∗^) or (control trial^∗^) or (clinical trials^∗^)):ti, ab, kw
10	Or 8–9
11	4 and 7 and 10

#### 2.4.2 Search for other resources

Aside from above electronic databases, we will also search conference proceedings, dissertations, and reference list of associated reviews.

### 2.5 Data collection and analysis

#### 2.5.1 Study selection

All searched records will be imported to the Endnote X7 and all duplicated data will be eliminated. Then, all titles and abstracts will be screened. After that, we will read full text of all potential papers to determine if they fit the final inclusion criteria. Any inconsistencies between 2 researchers will be worked out by a third researcher through discussion. We will note any exclusion reasons for all removed studies. The whole process of study selection will be demonstrated in a flow diagram.

#### 2.5.2 Data extraction and management

Two researchers will independently extract all relevant data from included studies using a standardized data extraction sheet. Any deviations between 2 researchers will be coped with a third researcher through consultation. The extracted information consist of study title, first author, country, time of publication, characteristics of patients, study setting, randomization, binding, allocation, concealment, sample size, details of interventions, controls, follow-up, outcome indicators, study results, adverse events, and funding information. If we identify any missing or unclear data, we will contact primary author to request them by email.

#### 2.5.3 Risk of bias assessment

Two researchers will independently appraise the risk of bias assessment for each qualified study using Cochrane risk of bias tool based on the guidelines of Cochrane Handbook for Systematic Reviews of Interventions. It covers 7 aspects, and each item is further referred as low, unclear, or high risk of bias. Any different opinions between 2 researchers will be disentangled by a third researcher through discussion.

#### 2.5.4 Measurement of treatment effect

For enumeration data, we will employ the results as risk ratio and 95% confidence intervals (95% CIs). For continuous data, we will exert the results as mean difference or standardized mean difference and 95% CIs.

#### 2.5.5 Assessment of heterogeneity

The heterogeneity of the study results will be determined through *I*^2^ test. *I*^2^ ≤ 50% suggests low level of heterogeneity, and a fixed-effects model will be utilized. *I*^2^ > 50% reveals a high level of heterogeneity, and a random-effects model will be applied.

#### 2.5.6 Data synthesis

RevMan 5.3 software will be employed for statistical analysis in this study. Meta-analysis will be undertaken if we identified low level of heterogeneity, and sufficient data are collected on the similar characteristics of study and patient, treatments, controls, and outcome records. Otherwise, if high level of heterogeneity is found, we will perform subgroup analysis to detect the possible reasons for such situation. Moreover, we will carry out narrative summary for the study results by reporting detailed written commentary to present the target patient characteristics, study findings, types of treatments and controls, and outcomes.

#### 2.5.7 Reporting bias

Funnel plot and Egger regression test will be performed to explore any potential reporting bias if sufficient studies are available (normally at least 10 qualified studies).^[26]^

#### 2.5.8 Subgroup analysis

We will implement subgroup analysis according to the different characteristics of study or patient, intervention, comparators, and outcomes.

#### 2.5.9 Sensitivity analysis

We will preside over sensitivity analysis to explore the robustness of outcome results by excluding low-quality studies.

#### 2.5.10 Grading the quality of evidence

We will exploit Grading of Recommendations Assessment, Development and Evaluation method to check the strength of evidence for included study. Two researchers will independently manage all the assessments, and any different views between both of them will be solved by a third researcher through discussion.

## 3 Discussion

A variety of previous studies have reported that sigmoidectomy is often utilized in treating SCC. However, its results are still inconsistent. Thus, it is necessary and crucial to make sure whether sigmoidectomy is a good option for the treatment of patients with SCC. This study aims to systematically and comprehensively investigate the efficacy and safety of sigmoidectomy in treating SCC. The results of this study will provide helpful evidence for both clinical practice and future relevant studies.

## Author contributions

**Conceptualization:** Hua-ping Hou, Pu-guo Gui.

**Data curation:** Hua-ping Hou, Pu-guo Gui.

**Formal analysis:** Hua-ping Hou, Pu-guo Gui.

**Investigation:** Pu-guo Gui.

**Methodology:** Hua-ping Hou, Pu-guo Gui.

**Project administration:** Pu-guo Gui.

**Resources:** Hua-ping Hou.

**Software:** Hua-ping Hou.

**Supervision:** Pu-guo Gui.

**Validation:** Hua-ping Hou, Pu-guo Gui.

**Visualization:** Hua-ping Hou, Pu-guo Gui.

**Writing – original draft:** Hua-ping Hou, Pu-guo Gui.

**Writing – review & editing:** Hua-ping Hou, Pu-guo Gui.
